# Research on time series prediction of microclimate in agrivoltaic systems based on the long short-term memory and attention mechanism

**DOI:** 10.3389/fpls.2026.1755040

**Published:** 2026-02-03

**Authors:** Long Zhang, Jianhui Gong, Cuinan Wu, Erik Harry Murchie, Alexandra Jacquelyn Gibbs, Bingbing Liu, Chen Yang, Guijun Xu, Jinxin Zhang, Jiguang Guo, Maohua Xiao, Encai Bao

**Affiliations:** 1 College of Engineering, Nanjing Agricultural University, Nanjing, China; 2 Institute of Agricultural Facilities and Equipment, Jiangsu Academy of Agricultural Sciences, Nanjing, China; 3 Key Laboratory of Agricultural Engineering of the Middle and Lower Reaches of the Yangtze River, Ministry of Agriculture and Rural Affairs of the People’s Republic of China, Nanjing, China; 4 School of Biosciences, University of Nottingham, Nottingham, United Kingdom; 5 Shenzhen Energy Nanjing Holding Co., Ltd., Nanjing, China; 6 China Electric Power Engineering Consulting Group Co., Ltd., Beijing, China

**Keywords:** agrivoltaics, microclimate prediction, attention mechanism, long short-term memory, solar radiation, air temperature

## Abstract

**Introduction:**

Agrivoltaic (AV) systems combine photovoltaic (PV) power generation with agriculture to enhance land use and energy production. However, accurately predicting the microclimate within AV systems remains a challenge, primarily due to existing models failing to get their inherent temporal and spatial variability.

**Methods:**

To address this, this study used long short-term memory (LSTM) networks to process time-series data and incorporated an attention mechanism to adjust the importance of temporal features. The model considered two environmental parameters, including solar radiation intensity and air temperature. Data collected from experimental AV systems with different PV panel density in Nanjing, China. The performance of the LSTM-Attention model was compared with traditional machine learning methods and standard LSTM models.

**Results:**

The results demonstrated that the LSTM-Attention model outperformed the other models in predicting both solar radiation intensity and air temperature within AV systems with different PV panel density. Specifically, the Root Mean Square Error (RMSE) for radiation intensity predictions decreased by 28.0%, 35.7%, and 42.1% at different coverage densities. For air temperature predictions, the RMSE dropped by 39.0% in summer and 18.1% in winter. Importantly, the LSTM-Attention model maintained stable prediction performance even in winter and rainy weather conditions.

**Discussion:**

The results indicated that the LSTM-Attention model could effectively captured the complex temporal variations in solar radiation and air temperature within AV systems, especially under varying weather conditions. The study provides theoretical support for improving crop management strategies within AV systems.

## 1 Introduction

Agrivoltaics is an emerging agricultural model that combines photovoltaic (PV) power generation with farming, allowing for more efficient use of land (Brown, 2018; Stid et al., 2025). In recent years, agrivoltaics (AV) systems have become increasingly popular as a way to combine renewable energy generation with farming (Li et al., 2022a). They have been given a lot of attention from researchers and businesses due to their dual environmental and economic benefits (Chopdar et al., 2024).

In AV systems, microclimate have a direct impact on the photosynthetic efficiency and physiological metabolic processes of crops, affecting the productivity and benefits of the system (Ghosh, 2023; Moon and Ku, 2023). The intensity of solar radiation that drives the process of photosynthesis is crucial for determining the rate of dry matter production and the final quality parameters of the crop (Gnayem et al., 2024). Air temperature also affects the activity of enzymes and respiration in plants, controlling the growth cycle and stress resistance performance (Gong et al., 2025). It has been found out that the shading caused by the PV panel results in significant changes in the radiation intensity both in space and time, and these changes in the light environment led to complex temperature field changes via alterations in the surface energy balance. All these together can cause physiological problems such as photoinhibition or heat stress for crops. As a non-linear system that is influenced by various factors such as the angle of the sun, clouds, and the shape of the PV array, AV systems display clear temporal and spatial variations in their microclimatic parameters (Pandey et al., 2025). To get a good grasp of the coupling among solar radiation and air temperature and to build up precise prediction models would offer some help to make decisions concerning crop cultivation and at the same time it would also form the basis for designing PV arrays and intelligent control systems (Zainali et al., 2025). Although environmental prediction for PV greenhouses is relatively well-established, there is still limited research on environmental prediction in open-field AV systems. Open-field AV systems differ significantly from greenhouse setups, primarily due to the lack of physical enclosures and the direct interaction between crops and external environmental factors such as wind, precipitation, and solar radiation. Consequently, accurately predicting microclimates in these systems is critical for optimizing both agricultural production and energy generation. By improving the prediction of environmental factors such as solar radiation and air temperature, it could provide data-driven insights that can assist in designing more efficient PV panel layouts and management strategies for crop cultivation.

The current methods used for predicting microclimates in AV systems include three main types of modeling methods: mechanistic approach, computational fluid dynamics (CFD), and data driven method (Li et al., 2021; Williams et al., 2023; Yajima et al., 2023). In practical applications, PV modules block incoming solar radiation in AV systems, forming moving shadows on the ground surface such as farmland, which affects the microclimate. Therefore, mechanistic models generally use solar radiation theory or thermodynamics to study the light distribution and dynamic energy-mass exchange process in these systems. For example, Ma Lu et al. (2022) developed a photosynthetic active radiation (PAR) decomposition model according to the Spitters light response curve theory. By adding cloud cover improvement elements and satellite-based information, they decreased the prediction mistake of the diffuse PAR part to 23.75% at a 30-minute time scale in temperate regions. Zhang et al. (2025) created a spatiotemporal dynamic model for the PV shading width, allowing for the quantitative prediction of the shading effect. Peng et al. (2023) created a light-electricity-heat coupling multi-physics model for PV greenhouses. They achieved a dynamic match between the coverage of PV panels and the photosynthetic characteristics of strawberries by optimizing the PAR and net photosynthetic rate models together. Some parameters are quite hard to get right, such as convective heat transfer coefficients and longwave radiation exchange coefficients. Additionally, the effect of crops on the surrounding environment continues to be a topic of active research, making it challenging to develop complete mechanistic models for AV systems (Zhang et al., 2025).

The CFD model is a special type of mechanistic models (Bellone et al., 2024). Recently, CFD modeling approaches have been extensively employed in studies of AV systems. A representative study by Joseph et al. (2025) implemented coupled multi-physics simulations incorporating source-sink terms within a CFD framework to analyze microclimates in AV systems. The study reduced numerical diffusion artefacts in shaded obstacle simulations through the refinement of the discrete ordinates radiation method. Williams et al. (2023) conducted a comprehensive CFD-based microclimate analysis examining the coupled effects of panel elevation, surface reflectivity, and plant water loss in AV systems. Gong et al. (2025) applied a CFD thermal environment model to explore the thermal regulation in AV systems with different panel heights. While these established CFD models can accurately simulate microclimate distribution within AV systems, they require substantial computational resources. In conclusion, CFD models are better suited for applications that do not have high real-time computation requirements such as agricultural buildings’ structural optimization and energy consumption simulation, rather than real-time environmental control in AV systems (Zainali et al., 2025).

The rapid development of artificial intelligence and computing technologies has prompted increasing adoption of data-driven models for microclimate prediction (Li et al., 2022b; Huang et al., 2024; Li et al., 2025). Although the training process demands considerable computational resources, these models achieve rapid prediction speeds post-training, with their accuracy being highly contingent upon both dataset quality and algorithmic design. Commonly employed machine learning approaches include regression models and convolutional neural networks (Giudici et al., 2023). Among these, the long short-term memory (LSTM) have emerged as a prevalent algorithm for time-series processing (Wu et al., 2024), finding particular application in agricultural building microclimate prediction due to their superior capability in handling sequential data (Geng et al., 2024). However, as the forecasting horizon extends, there is a significant decline in prediction accuracy, making the achievement of precise microclimate predictions under complex scenarios a key research challenge.

As a major innovation for processing sequence data, attention mechanisms dynamically allocate focus among inputs to enhance the model’s capability to recognize and leverage important information (Guo and Feng, 2024). Recent advances have demonstrated its effectiveness in enhancing model predictive performance. Mao et al. (2024) integrated attention mechanisms into BiGRU networks; the computed attention weights for hidden state vectors greatly enhanced the extraction of temporal features in greenhouse environments. Jiang et al. (2022) introduced an Attention-LSTM architecture which yielded impressive results in predicting indoor temperature 90 minutes ahead. They used a four-head attention mechanism to dynamically weight important features such as past temperatures, exterior wall temperatures, and predicted outdoor temperatures, resulting in a coefficient of determination of 0.9, which is a 26.1% improvement over traditional LSTM methods. However, research on applying attention mechanisms to enhance model predictive performance in AV systems remains relatively unexplored.

PV panel coverage density, which is an important structural parameter affecting the microclimate of AV systems, directly affects the spatial distribution patterns and dynamic changes of photothermal resources. The shading effect of PV panels with different coverage densities greatly changes the surface energy balance, causing spatiotemporal differences in the radiation intensity and temperature field, thereby affecting the photosynthesis efficiency and physiological metabolic process of crops (Tan et al., 2025). To conduct microclimate parameter prediction research on multiple density scenarios can not only clarify the quantitative response relationship between the coverage of PV panels and the microenvironmental factors, but also provide data support for optimizing the configuration of PV arrays, so as to achieve the goal of improving the efficiency of power generation and agricultural production at the system level.

To deal with the considerable temporal variation and spatial disparity of microclimate parameters in AV systems, and the reduced predictive precision with longer forecasting intervals, this research created an LSTM-Attention based multi-parameter microclimate prediction model. The model used a multi-dimensional environmental parameter combination such as the PV panel coverage density and photothermal environment and adopted an attention mechanism to dynamically weight important temporal factors. This method was able to make highly accurate forecasts of the microclimate in AV systems during different seasons and weather conditions, which provided a theoretical basis for optimizing the allocation of light and thermal resources and managing crops.

## 2 Materials and methods

### 2.1 Experimental AV systems

**Figure 1:**
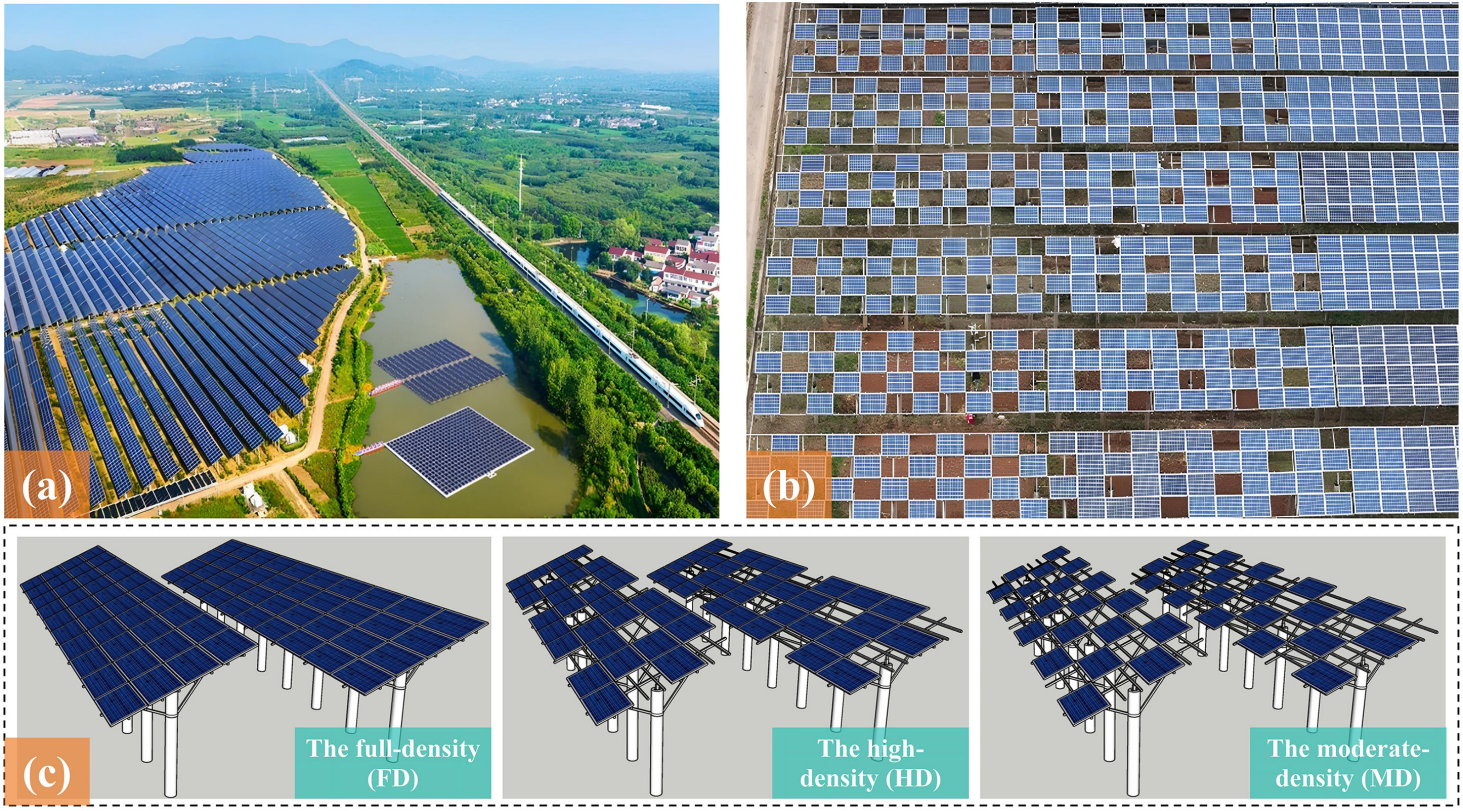
Location of the AV systems and different structure with 3 coverage densities: **(a)** Experimental AV base; **(b)** PV array structure; **(c)** Arrays with 3 different coverage densities of the moderate-density (MD), the high-density (HD) and the full-density (FD).

The experimental AV park located in Nanjing, China (31.6°N, 119.2°E). This park covered a total area of 47 hectares at an altitude of 360 meters, which was constructed in 2016 by Shenzhen Energy Group. Operating under an AV model, it comprised eight array units (**Figure 1a**). The park was estimated to reduce CO2 emissions by 19,800 t per year, which is equivalent to saving 7,900 t of standard coal. estimated to reduce CO_2_ emissions by 19,800 t per year, equivalent to saving 7,900 t of standard coal. The experimental area has a subtropical monsoon climate with obvious seasonal changes.

The AV system adopted a south-facing array configuration with a total span of 6.8 m. Under full-density coverage, the PV modules were arranged in four tightly packed horizontal rows (**Figure 1b**). The modules were mounted at a 24° tilt angle on *Φ*300 concrete piles, with the lower edge positioned 2.5 m above ground level. The polycrystalline silicon PV modules had a rated power output of 265 Wp, with a dimension of 1640 × 992 × 35 mm (L × W × H). Considering that low PV array coverage would result in impractical investment recovery periods while accounting for retrofitting costs, the study established two typical PV coverage densities of the moderate-density (MD) and the high-density (HD), based on a tightly packed configuration of 4 horizontal rows representing the full-density (FD) (**Figure 1c**). The vertical projection ratios of these coverage levels (the total vertical projection area of the modules on the farmland/the area of the farmland where the photovoltaic modules are installed) are 53.3%, 40.0% and 26.7% respectively.

### 2.2 Data acquisition

To investigate the shading effects of PV arrays, varying PV panel deployment densities (full-density, FD; high-density, HD; and moderate-density, MD) were employed to establish quantitative relationships between shading ratios and microenvironmental parameters, thereby comprehensively covering typical operational scenarios. The monitored AV system in this study comprised four distinct sensor monitoring zones: three inter-panel areas within AV systems with different panel coverage densities and an open-field area without PV panels. The environmental parameters monitored included global solar radiation, temperature, relative humidity, soil moisture, precipitation, wind speed and direction, both within and outside the AV systems. Detailed specifications of the sensor models and their parameters are provided in **Table 1**. The data recording system integrated a compact weather station recorder, equipped with solar radiation sensors, temperature sensors and other instruments.

**Table 1:** Details of specifications of sensors for environmental data collection.

Type	Manufacturer	Model	Measured variable	Accuracy
Air temperature and humidity sensor	Onset Co., Ltd., USA	HOBO UX100-011A	Air temperature Relative humidity	± 0.2°C ± 2.5%
Total solar radiation intensity sensor		HOBO S-LIB-M003	Solar radiation intensity	± 10 W/m^2^
Temperature sensor		HOBO S-TMB-M006	Soil temperature and PV panels temperature	± 0.2°C
Soil moisture sensor		HOBO S-SMC-M005	Soil moisture	± 3.1%
Small weather station data recorder		HOBO H21-USB	—	—
Rain gauge cylinder	Shandong Jianda Renke Electronic Technology Co., LTD., China	RS-YL-6	Precipitation	0.5 mm
Wind speed and direction sensor		RS-FSJT-N01 RS-FXJT-360	Wind speed and direction	0.1 m/s 360°

**Figure 2:**
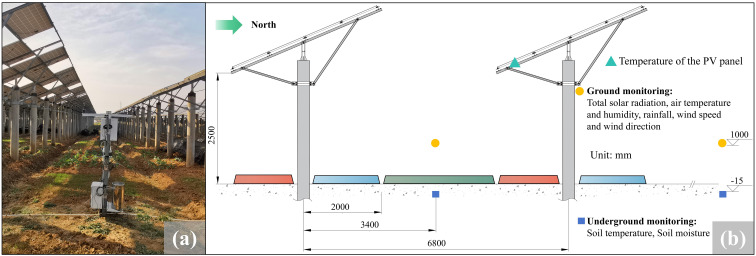
Monitoring scene and experimental measuring point: **(a)** Monitoring scene; **(b)** Arrangement of the experimental measurement points.

As shown in **Figure 2a**, all sensors were placed for long-term, continuous monitoring at intervals of 10 min. The sensor layout followed the following principles: Under the condition of meeting the information collection requirements, reduce the redundancy of sensor configuration; Taking into full account factors such as shading of PV modules, plant height, and root length of plants, the expected test results can fully reflect objective laws. The spatial distribution of sensor locations was presented in **Figure 2b**. The data collection period spanned from June 15, 2023, to June 15, 2024, which covered an entire year, including all four seasons. Specifically, the data collection included critical periods such as the summer solstice, with the training set from June 15, 2023, to July 5, 2023, and the validation set from July 16, 2024, to July 24, 2023, representing high-temperature and high-radiation conditions. The winter solstice, with the training set from December 8, 2023, to December 28, 2023, and the validation set from December 29, 2023, to January 6, 2024, representing low-temperature and low-radiation conditions. In addition, we selected data from various weather conditions to enhance the model’s robustness. The sensors operated continuously with 10-minute intervals to monitor parameters. The experimental protocol set daytime as 06:00-18:00 and nighttime as 18:00-06:00^+1^ (Zhang et al., 2023). According to the observation of cloudiness, precipitation record and sunshine duration measurement, the weather condition during the study period was divided into three types, clear sky, overcast and rainy condition (Zhang et al., 2025).

### 2.3 Data preprocessing

#### 2.3.1 Missing values

In order to make sure that data imputation can be feasible and to prevent long-distance false interpolation due to too much missing data, this study used box plots to remove outliers from the collected data. Then, the missing values were filled with mean imputation, linear interpolation, and data from the closest position with similar weather conditions (Ritter, 2023).

#### 2.3.2 Normalization

To ensure data consistency and improve processing efficiency, the study normalized all the input environmental elements to the interval [0, 1]. Normalization of the input data *X*_1_, *X*_2_, *X*_3_… *X*_N_ was performed by Equation 1 (Singh and Singh, 2020; Yang et al., 2023).

(1) $$X=\;\frac{X_{i}-X_{\text{min}}}{X_{\text{max}}-X_{\text{min}}}$$

Where, *X*_i_ is the original data, *X*_min_ is the minimum values of the original dataset, *X*_max_ is the maximum values of the original dataset, and *X* is the normalized value after transformation.

#### 2.3.3 Correlation analysis

The collected environmental data were analyzed by the Pearson correlation coefficient (PCC) method. The PCC measured the linear correlation of two continuous random variables, as shown in Equation 2. The PCC ranges from -1 to 1, where values approaching 1 indicate stronger correlations between variables (Jiang et al., 2025).

(2) $$R=\frac{\sum_{k=1}^{p}(X_{k}-\bar{X})(Y_{k}-\bar{Y})}{\sqrt{\sum_{k=1}^{p}{\left(X_{k}-\bar{X}\right)}^{2}}\sqrt{\sum_{k=1}^{p}{\left(Y_{k}-\bar{Y}\right)}^{2}}}$$

Where, *X_k_* and *Y_k_* are the *k*-th feature variable and environmental sample data, *p* is the number of samples, $\bar{X}$ and $\bar{Y}$ are the mean values of the feature variables and the environmental data from the AV systems.

#### 2.3.4 Data preparation

**Figure 3:**
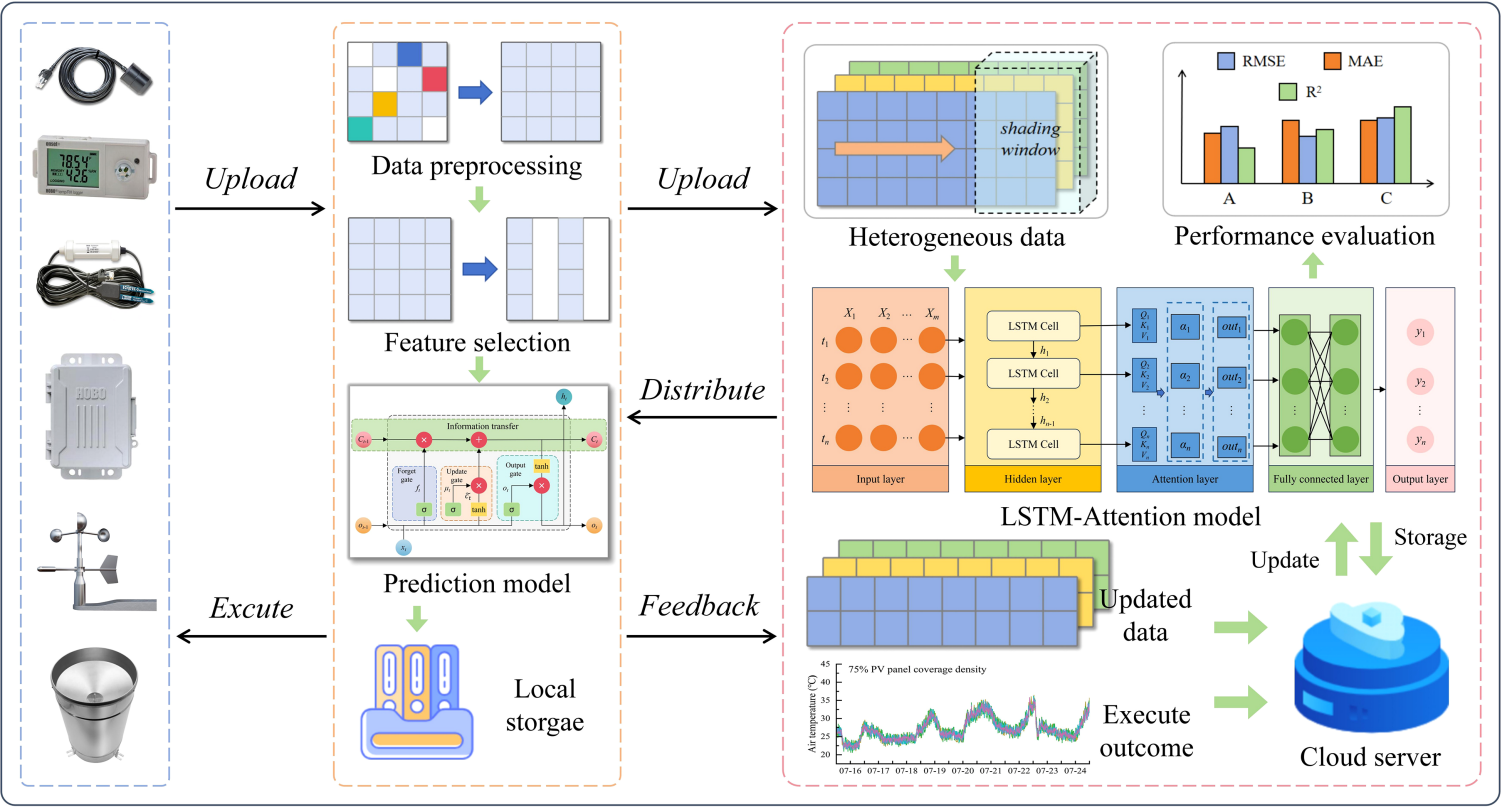
Microclimate prediction process.

To rigorously evaluate model robustness under extreme climatic conditions, we specifically selected data from 30-day periods centered around the summer solstice (representing high-temperature, high-radiation conditions) and winter solstice (representing low-temperature, low-radiation conditions) for model training and validation. The dataset was partitioned such that each seasonal subset contained 21 consecutive days (70% of data) for training and 9 consecutive days (30%) for validation. The sensor records every 10 minutes, and a single environmental parameter generates 1,008 measurements per day. For a single environmental parameter, the training set for each season contained 3,024 environmental data points (21 days), and the validation set contains 1,296 data points (9 days). The whole process of data processing and microclimate prediction of AV systems in this study was shown in **Figure 3**.

## 3 Models for solar radiation and air temperature prediction

### 3.1 Long short-term memory

**Figure 4:**
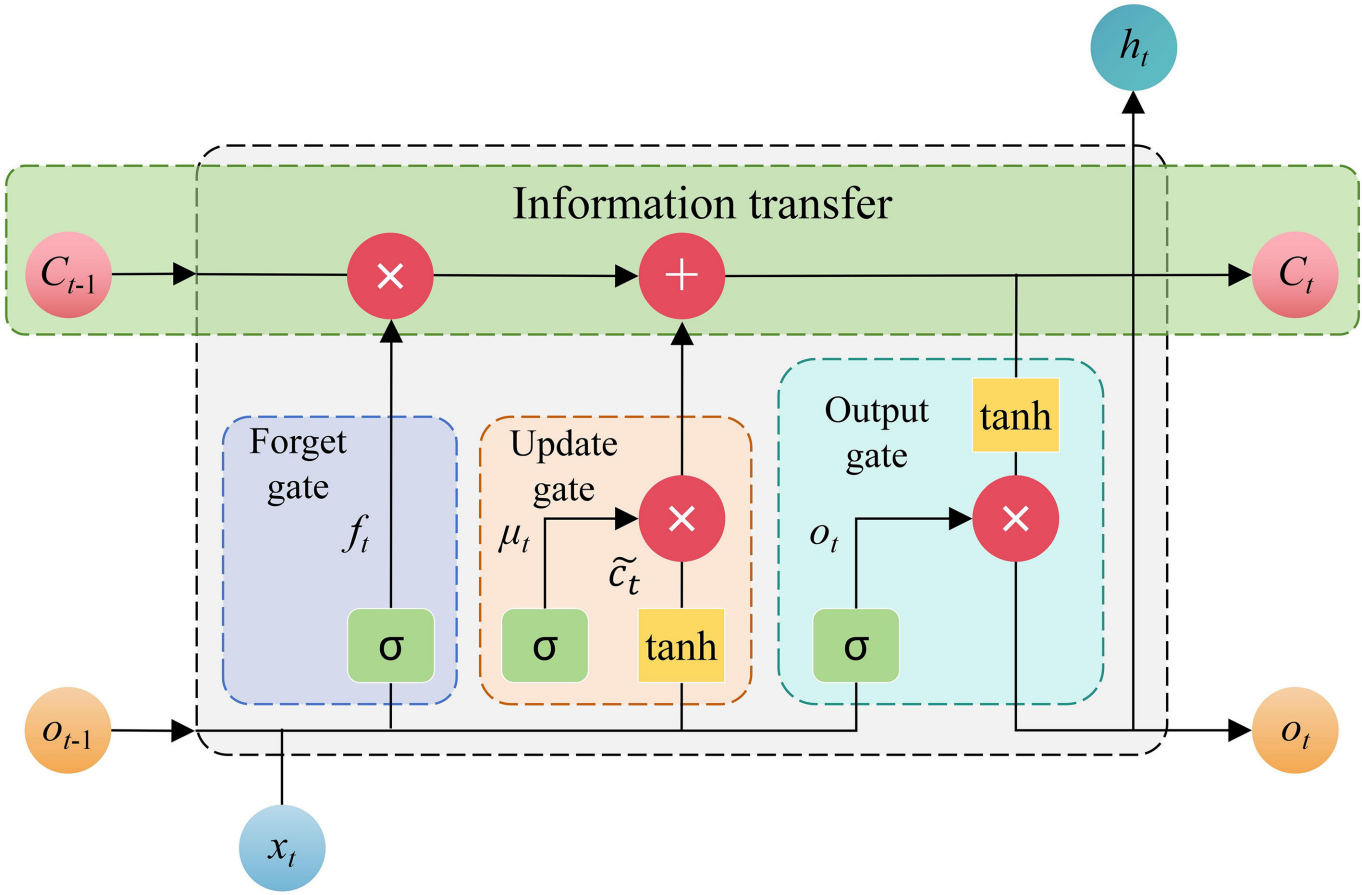
Door structure of long short-term memory. *C_t_*_-1_ is cell state at time *t*-1, *C_t_* is cell state at time *t*, *h_t_*_-1_ is output at *t*-1, *h_t_* is output at *t*, *f_t_* is forget gate, *μ_t_* is update gate, *o_t_* is output gate, $\tilde{c_{t}}$ is cell state, *σ* is activation equation and tanh is hyperbolic tangent function.

Microclimate variables in agrivoltaic systems exhibit strong temporal dependence, long-range dependencies, and nonlinear fluctuations due to moving shadows, cloud dynamics, and seasonal changes, which calls for a time-series model that can robustly learn long sequences. LSTM, with its gated architecture, effectively mitigates vanishing/exploding gradients in long-sequence modeling and is therefore well-suited for capturing long-term temporal patterns (Hochreiter and Schmidhuber, 1997; Landi et al., 2021). The LSTM architecture has a complex gate mechanism for controlling the flow of information and updating cell states, thus controlling both information storage and transmission. As shown in **Figure 4**, the LSTM gating system comprises four fundamental units. At each timestep *t*, the model processes 3 inputs: (i) the external input at *t*, (ii) the LSTM unit output at *t*-1, and (iii) the cell state at *t*-1. These inputs undergo transformation through the gating units to produce updated outputs and cell states (Fang et al., 2021). The mathematical formulations governing these operations were provided in Equations 3–8.

(3) $$f_{t}=\sigma (W_{f}\cdot [h_{t-1},\;x_{t}]+b_{f})$$

(4) $$i_{t}=\sigma (W_{\mu }\cdot [h_{t-1},\;x_{t}]+b_{\mu })$$

(5) $${\tilde{c}}_{t}=\tanh(W_{c}\cdot [h_{t-1},\;x_{t}]+b_{c})$$

(6) $$C_{t}=f_{t}\cdot C_{t-1}+i_{t}\cdot {\tilde{C}}_{t}$$

(7) $$o_{t}=\sigma (W_{o}\cdot [h_{t-1},\;x_{t}]+b_{o})$$

(8) $$h_{t}=o_{t}\cdot \tanh(C_{t})$$

Where, tanh denotes the hyperbolic tangent function and *σ* represents the activation function. The weight matrices (*W_f_*, *W_μ_*, *W_c_*, *W_o_*) and bias vectors (*b_f_*, *b_u_*, *b_c_*, *b_o_*) correspond to the forget gate, update gate, cell state, and output gate, respectively. These parameters were initialized randomly and subsequently optimized through data-driven training.

### 3.2 Attention mechanism

The attention mechanism comes from animal’s visual attention. This mechanism enables models to concentrate more on the most important information from the input sequence, which makes it better at recognizing important features and improves its prediction accuracy. The attention mechanism dynamically assigns higher weights to the most informative time steps, enabling the model to focus on critical information while suppressing noise, thereby improving responsiveness and accuracy under rapidly varying shading (Niu et al., 2021).

**Figure 5:**
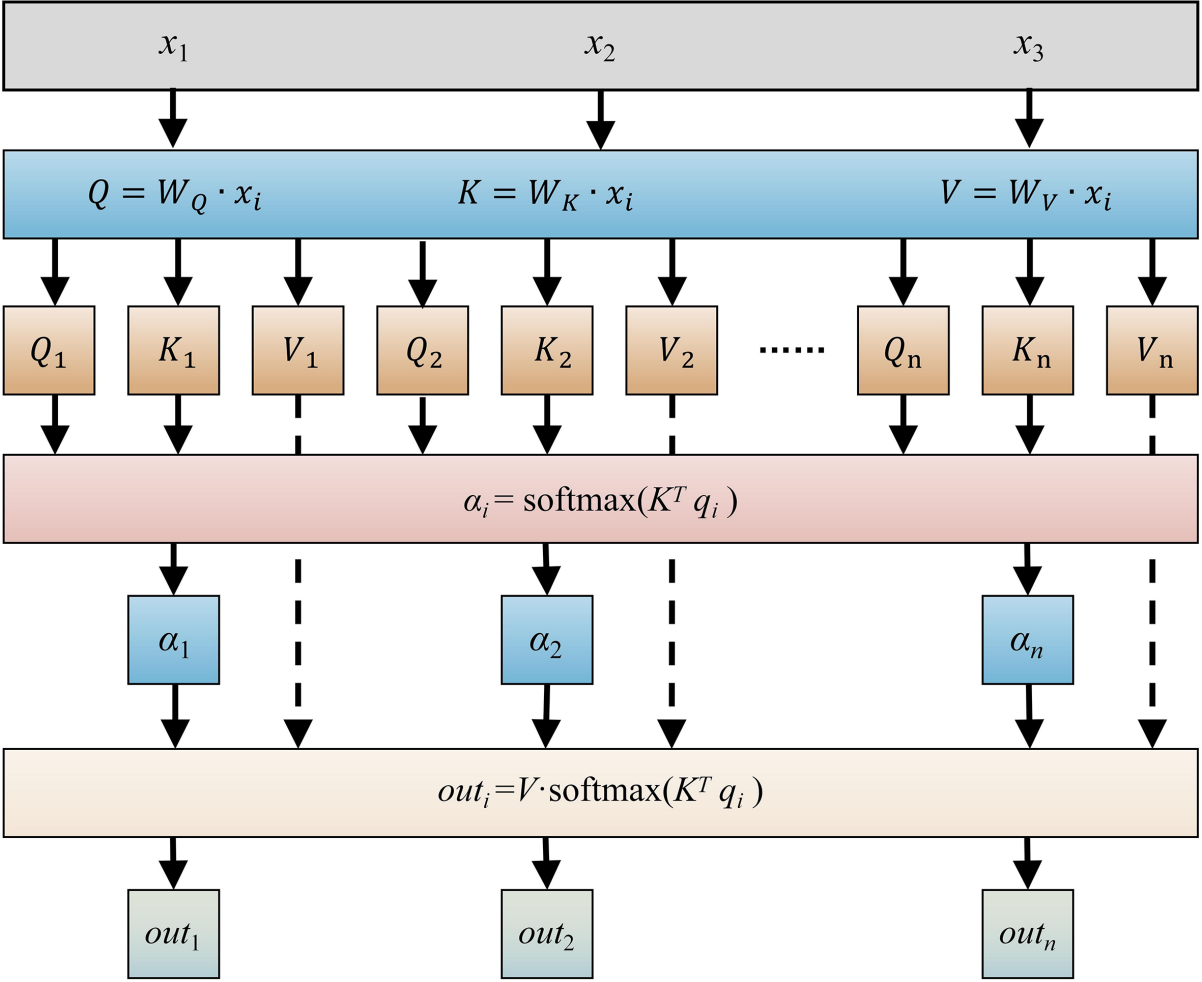
Schematic diagram of attention mechanism. *x*_1_, *x*_2_, *x_n_* are feature input; *W_Q_*, *W_K_* and *W_V_* are parameter matrix; *Q* is query vector, *K* is key vector, *V* is value vector; *q*_i_ is the element of *Q* vectork, *k*_i_ is the element of *K* vector, *v_i_* is the element of *V* vector; *α_i_* is the attention distribution; Softmax is the normalized function; *out_i_* is output of attention mechanism; *i* is the feature number, the range is 1 to *n*.

The attention mechanism works by taking three input vectors: query (*Q*), key (*K*), and value (*V*). The vector *Q* gets relevance scores with other vectors, while the key vector *K* measures the connection strength between *Q* and other vectors. The value vector *V* contains the information to be aggregated according to the attention weights gotten from *Q*. The mechanism calculates the similarity between *Q* and *K* to determine how much attention should be given to each element in *V* during the weighted aggregation process. Specifically, it first calculates the dot product of *Q* and *K*, applies a normalization operation to get the importance weights of each element in *K*, and then performs a weighted sum of *V* with these weights to get the final representation. As shown in **Figure 5**, the attention computation has three main steps (Gao et al., 2022).

Step 1: The query (*Q*), key (*K*), and value (*V*) matrices were computed using Equations 9–11.

Step 2: The similarity scores between *Q* and *K* were calculated via Equation 12. These scores were then normalized using the softmax function to obtain attention weights, where higher values indicate stronger relevance between the i-th input and the task objective.

Step 3: The final attention output was generated by performing a weighted sum of *V* with the normalized attention weights, as defined in Equation 13.

(9) $$Q=W_{Q}*x_{i}$$

(10) $$K=W_{K}*x_{i}$$

(11) $$V=W_{V}*x_{i}$$

(12) $$\alpha_{i}=softmax(K^{T}q_{i})$$

(13) $$c_{i}=V\cdot softmax(K^{T}q_{i})$$

### 3.3 Long short-term memory – attention

**Figure 6:**
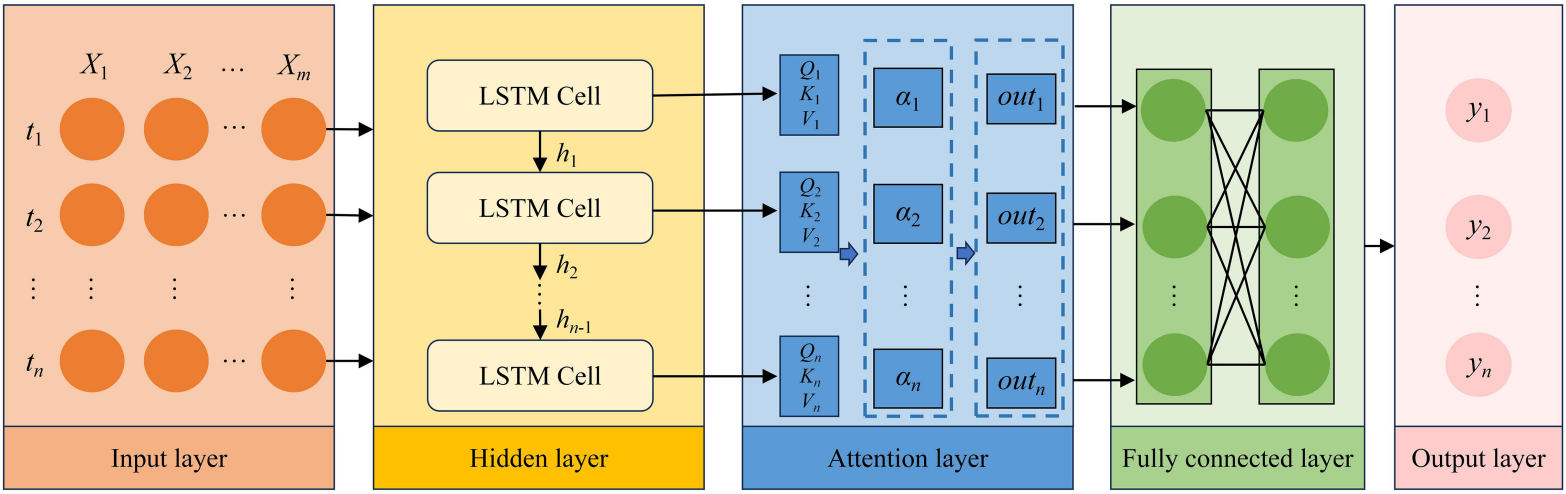
Network structure of LSTM-Attention prediction model. *t*_1_, *t*_2_,…, *t_n_* are time series; *X*_1_, *X*_2_,…, *X_m_* are input variables; LSTM is long short-term memory; *y*_1_, *y*_2_,…, *y_n_* are output.

To enhance the prediction of microclimate within AV systems, the study developed an integrated LSTM-Attention model. The framework of the model was illustrated in **Figure 6**. The model architecture operates as follows: First, multivariate time-series data were processed through an LSTM layer for feature extraction. Subsequently, an attention mechanism was applied to compute weight coefficients, which quantify the relevance between current and historical data points. By adaptively assigning higher weights to more informative temporal features, the model effectively captured the most critical patterns while suppressing noise. The structure of the LSTM-Attention model includes 5 layers (Ma et al., 2024). The description of each layer is given below:

1. Input Layer: Multidimensional time-series features are converted to LSTM-compatible 3D arrays (*S*, *T*, *X*). Where, *S* is the number of input samples, *T* is the time dimension, and *X* is the feature dimension.
2. LSTM Layer: Two LSTM layers are interlaced with dropout layers to prevent overfitting by stochastically deactivating neurons during training, which improves generalization ability.
3. Attention layer: To compute the attention mechanism, we used Equations 9–11 to derive the *Q*, *K*, and *V* vectors for each hidden state. The similarity scores between *Q* and *K* were then calculated through Equation 12, followed by normalization via the Softmax function to obtain the attention distribution. Higher values in this distribution indicate stronger relevance between the input information and task objectives. Finally, the attention output was computed as the dot product between the normalized weights and the value vectors (Equation 13).
4. Fully Connected Layer: Attention outputs were processed through dense connections for feature recombination and dimensional transformation.
5. Output Layer: Final predictions were generated with linear activation.

Since the target subjects, regional climatic characteristics, as well as the feature variables, data volume, and data quality of the dataset in this study differ from those in previous studies, it would lack scientific rigor to compare and discuss the predictive performance of the model in this study with that of previous research. To assess the improvement of the LSTM-Attention model over the standard LSTM model and several widely used machine learning models in microclimate prediction, the study conducted comparative analyses under two scenarios: varying prediction time horizons and different weather conditions. Specifically, the LSTM-Attention model was compared with four commonly employed machine learning models—BP, SVM, LSTM, and LSTM-GRU—in terms of their predictive performance for both solar radiation intensity and air temperature within AV systems.

### 3.4 Algorithm parameter settings

The internal environmental factor model of the AV system was developed using Python 3.11, with PyCharm as the development environment and TensorFlow as the development framework. The training process involved 80 iterations, a learning rate of 0.01, and an input batch size of 32, with the maximum number of epochs set to 100. The “Adam” optimizer and “mean squared error” loss function were employed for model training. Additionally, the number of neurons in both the GRU and LSTM layers was set to 128. To maintain consistency in model comparisons, all modules across were configured with the same parameters.

The study selected several machine learning models for comparison, including BP, SVM, and LSTM-GRU. The rationale for choosing these models was based on their ability to handle sequential data and their inherent differences in handling long-term dependencies. However, these models still faced challenges when dealing with the complex temporal and spatial variations in agrivoltaic systems, particularly under changing weather conditions.

### 3.5 Evaluation indicators

To assess the model’s performance, the study utilized the coefficient of determination (*R*^2^), root mean square error (RMSE), and mean absolute error (MAE). The mathematical expressions for these metrics were given in Equations 14–16.

(14) $$R^{2}=1-\frac{\sum_{k=1}^{n}{\left(X_{k}-Y_{k}\right)}^{2}}{\sum_{k=1}^{n}{\left(\bar{Y_{k}}-Y_{k}\right)}^{2}}$$

(15) $$RMSE=\sqrt{\frac{1}{n}\sum_{k=1}^{n}{\left(X_{k}-Y_{k}\right)}^{2}}$$

(16) $$MAE=\frac{1}{n}\sum_{k=1}^{n}|\frac{X_{k}-Y_{k}}{Y_{k}}|\times 100\%$$

Where, *X_k_* is the predicted value, *Y_k_* is the observed value, $\bar{Y_{k}}$ is the mean of the observed values, and *n* is the total number of predicted samples.

## 4 Results and discussions

### 4.1 Correlation analysis

**Figure 7:**
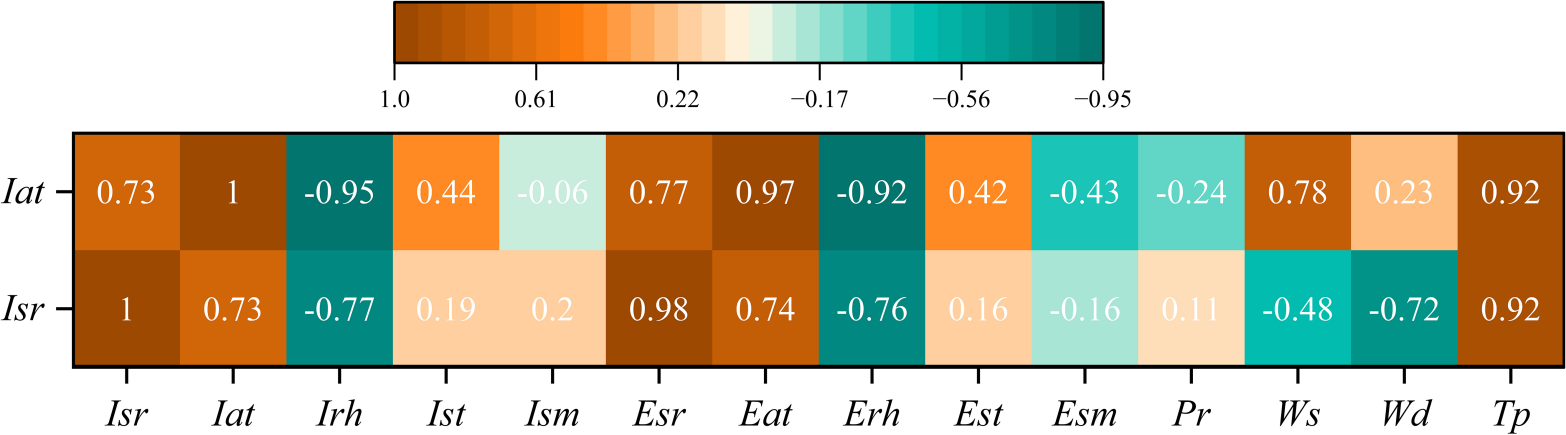
Pearson correlation between the solar radiation (*Isr*) and air temperature (*Iat*) inside AV systems with different environmental factors. *Isr* represents the intensity of internal solar radiation, *Iat* represents the internal air temperature, *Irh* represents the internal relative humidity, *Ist* represents the internal soil temperature, *Ism* represents the internal soil moisture, *Esr* represents the external solar radiation, *Eat* represents the external air temperature, *Erh* represents the external relative humidity, *Est* represents the external soil temperature, *Esm* represents external soil moisture, *Pr* represents precipitation, *Wd* represents the wind direction, *Ws* represents the wind speed, *Tp* represents the temperature of the PV panel.

Microclimate parameters in AV system have complex nonlinear interdependence with different environmental factors. Including all measured factors as model inputs can add noise and reduce the accuracy of predictions because of possible multicollinearity and interference from irrelevant features (Wang and Chen, 2021). **Figure 7** showed a complicated nonlinear relationship among the microclimate parameters inside the AV system, where solar radiation intensity and air temperature exhibited differential coupling with various environmental factors. According to Pearson correlation analysis, the internal solar radiation intensity was highly correlated with the PV panel temperature and the external solar radiation, but it had a moderate association with the relative humidity and soil temperature. External air temperature and wind speed showed considerable but less noticeable connections to the inner microclimate parameters, which implied that the atmosphere has a secondary effect on the inner environment through the PV array. These results matched the known rules about energy balance in partly closed farming places, where how the leaves are shaped and what they’re made of affect how they interact with their surroundings (Ali Abaker Omer et al., 2025).

For solar radiation intensity, the analysis showed very strong relationship with the external solar radiation and temperature of PV panel, while demonstrating moderate relationships with the external air temperature and internal relative humidity. There were weak correlations between the strength of the internal solar radiation and the moisture level of the soil or the direction of the wind. It suggested that external solar radiation and temperature of PV panel should be the main input variables for radiation prediction, and external air temperature and internal relative humidity could be added to consider the effect of atmospheric attenuation. Differential correlation pattern indicated that we need different features for the model of these two important microclimate parameters. The internal air temperature showed very high correlation with external air temperature and PV panel temperature. It exhibited a moderate correlation with relative humidity and soil temperature, while showing weak correlations with wind speed and precipitation. The results showed that the main factors affecting the prediction of air temperature were the external air temperature, temperature of PV panel, and internal relative humidity, and the internal soil temperature and wind speed should be considered as secondary.

### 4.2 Analysis of radiation intensity prediction results

#### 4.2.1 Seasonal variations in predicted radiation intensity

**Figure 8:**
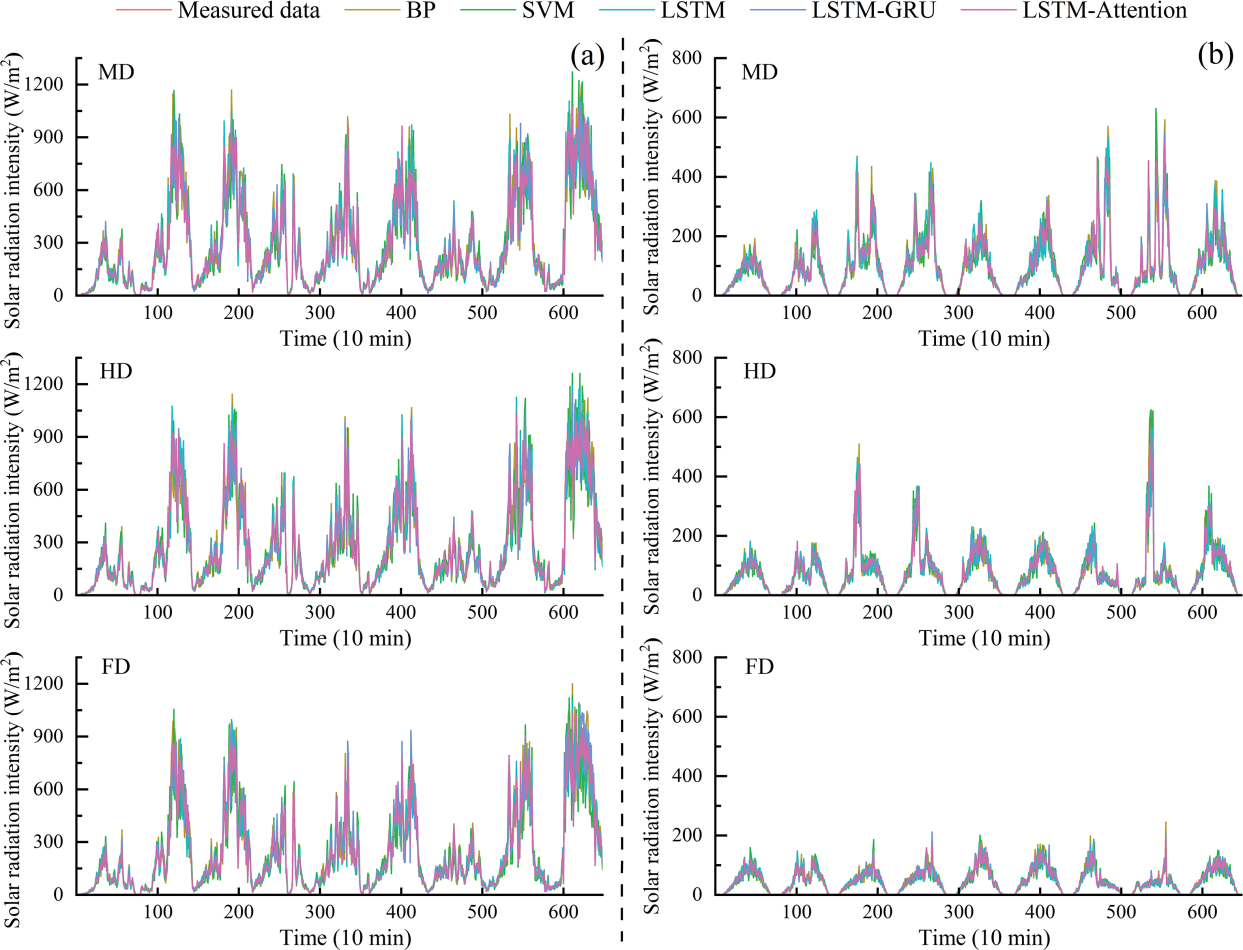
The estimated and measured value curve of solar radiation: **(a)** Summer; **(b)** Winter.

**Figure 8** presented the comparative curves of predicted and measured solar radiation intensity across different seasons for the BP, SVM, LSTM, LSTM-GRU, and LSTM-Attention models. The results revealed that the maximum prediction deviations for solar radiation intensity in summer under the MD, HD, and FD treatments for the five models were 300.4, 358.1, 252.9, 261.7, and 163.7 W/m^2^ (MD); 281.5, 344.3, 274.6, 232.9, and 173.4 W/m^2^ (HD); and 300.6, 343.0, 268.0, 233.2, and 167.4 W/m^2^ (FD), respectively. In winter, the maximum prediction deviations under the same coverage densities were 156.8, 160.0, 134.6, 116.9, and 80.0 W/m^2^ (MD); 165.9, 177.1, 169.1, 121.7, and 73.2 W/m^2^ (HD); and 54.3, 57.8, 73.7, 47.8, and 29.0 W/m^2^ (FD). These results demonstrated that the LSTM-Attention model achieved superior prediction accuracy for solar radiation intensity in both summer and winter, with its maximum deviation being significantly lower than those of the traditional machine learning models and the standalone LSTM or LSTM-GRU models.

The performance differences between LSTM and its improved versions, LSTM-GRU and LSTM-Attention, showed that improving the structure of the neural network could enhance the accuracy of forecasts in complicated energy areas. The LSTM model had a maximum prediction deviation of 252.9 W/m^2^ for summer solar radiation intensity under MD treatment. In comparison, the LSTM-Attention model reduced the maximum prediction error by 37.5% compared to the LSTM model under the FD treatment, demonstrating the quantitative analysis capability of the attention mechanism on multiple reflected radiations among PV panels. Furthermore, the LSTM model had a 25.6% decrease in the maximum prediction error for winter solar radiation intensity with HD treatment compared to MD treatment. However, its maximum error at the FD treatment was still higher than LSTM-GRU and LSTM-Attention. Notably, the LSTM-Attention model performed well in winter, it has the smallest maximum error in the whole dataset at the FD treatment, which is 60.7% less than that of the LSTM model. Regarding solar radiation intensity prediction as a particular environmental element, the LSTM-Attention model exhibited excellent seasonal adaptability. In the high summer radiation condition, the model could keep the prediction deviation below 170 W/m^2^ through the attention-weighted LSTM framework, which was over 50% better than the traditional machine learning model. In winter, under low-radiation conditions, its maximum deviation was only 29.0 W/m^2^ at the FD treatment, outperforming LSTM-GRU by 39.3%. In addition, it observed that higher PV coverage densities tend to increase error due to shading effects and localized temperature changes, which were harder for the model to capture. This was especially prominent in summer when radiation intensity fluctuations are more pronounced. This difference in performance showed that the attention layer improved important temporal features, and the LSTM part dealt well with the non-linear changes in radiation intensity (Kong et al., 2023; Şener and Tuğal, 2025).

Solar radiation intensity was a key microclimate parameter that was highly influenced by the density of the PV array. Our study showed that as the PV array density increased, shading effects became more pronounced, leading to greater spatial and temporal variation in radiation intensity. Specifically, under the HD and FD configurations, the model’s ability to predict solar radiation intensity decreased. During the summer, the increased shading caused by higher PV array densities resulted in significant fluctuations in radiation intensity, which the model had difficulty capturing. The RMSE increased with the array density, reflecting the growing complexity in predicting radiation under dense PV arrays. The model performed better under the MD configurations, where the shading effects were less severe. These findings indicated that the attention mechanism incorporated in the model helped mitigate the impact of shading to some extent, but further refinement was needed to address the more dynamic shading patterns in high-density configurations.

**Figure 9:**
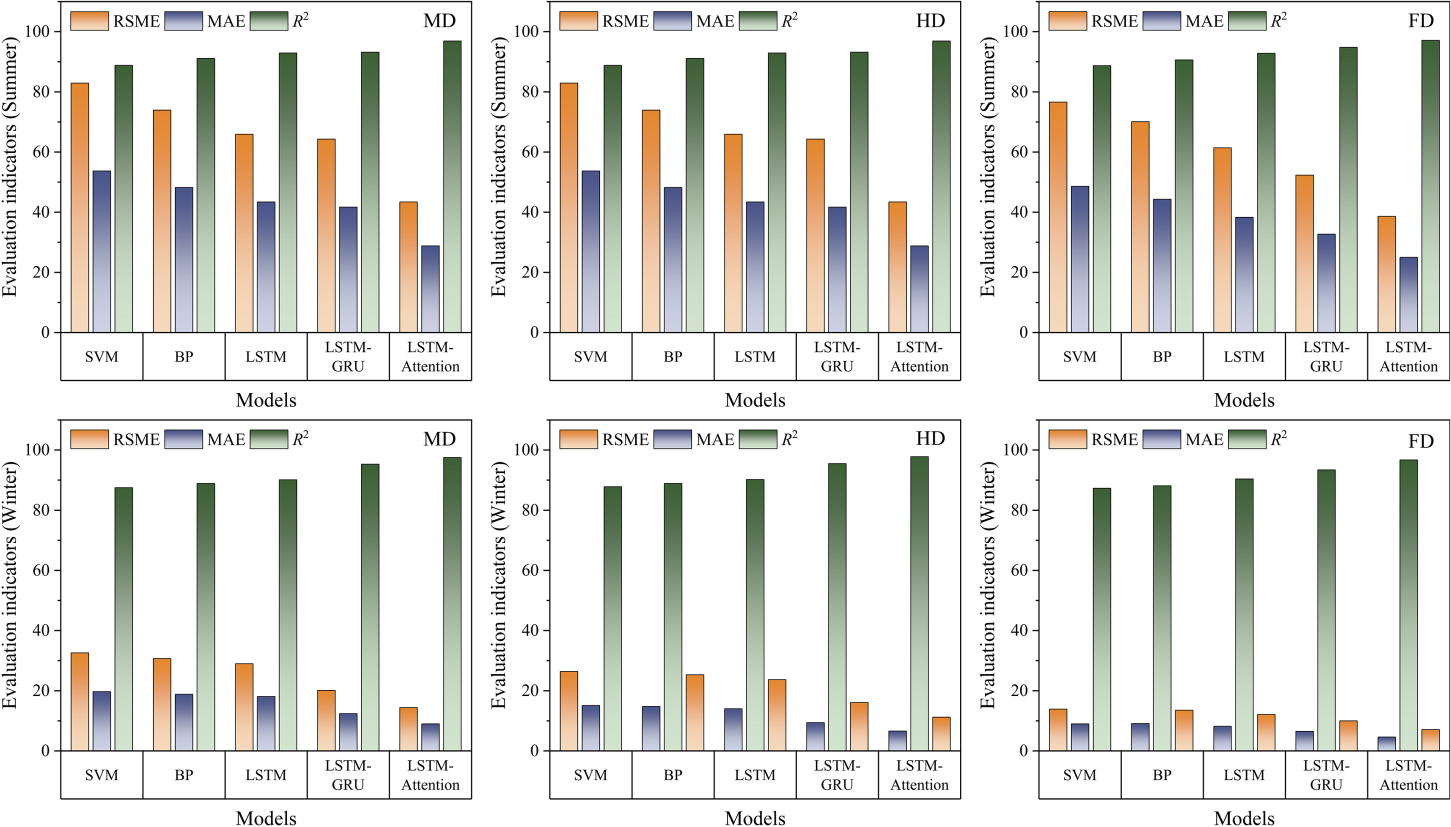
Performance comparison of five prediction models for solar radiation intensity in different forecasting seasons.

**Figure 9** showed the comparison of different solar radiation prediction models according to their seasonal variation and coverage density. Results show that the LSTM-Attention model did better than the others during both summer and winter. In summer, at MD, HD, and FD treatments, the LSTM-Attention model got a mean RMSE of 41.6 W/m^2^. It meant that the reduction was 28.0%, 35.7%, 42.1%, and 49.4% less than the LSTM-GRU, LSTM, BP, and SVM models, respectively. MAE had a similar trend, and *R*^2^ was around 96.9% to 97.1%, which meant it performed much better than other models. The benefit of LSTM-Attention was even more evident in winter. At the HD treatment, it reached an RMSE of 11.2 W/m^2^, which was 30.4%, 52.7%, 55.7%, and 57.6% lower than the LSTM-GRU, LSTM, BP, and SVM models, respectively. For this prediction, the *R*^2^ value was 97.8%, indicating that the model had a good ability to capture the temporal characteristics of solar radiation intensity.

Further analysis showed that the LSTM-Attention model had better stability than the benchmark models under changing environmental conditions. MD treatment RMSE was only 49.7% of the LSTM model’s RMSE in winter. As coverage density increased, the performance of the LSTM-Attention model decreased slightly; the *R*^2^ value dropped by only 0.8% at the FD treatment. In contrast, the *R*^2^ values of LSTM-GRU and LSTM decreased by 1.9% and 0.3%, respectively. It is likely because the attention mechanism can model the spatiotemporal variations in solar radiation intensity, particularly in winter when the fluctuations were caused by changes in the solar elevation angle. LSTM-Attention could make the key feature more important so as to avoid the sudden error of prediction. Traditional LSTM and LSTM-GRU models responded more slowly to sudden changes in radiation intensity compared to these models. This can be seen from the higher MAE values for these models in summer, which were up to 53.6% higher than the LSTM-Attention model under high coverage density conditions. From this, it can be seen that the mechanism reduced phase lag and peak mismatch during fast changing radiation period and improved robustness when the signal to noise ratio was low, which explained the stronger performance of LSTM-Attention under complex climate conditions.

#### 4.2.2 Out-of-window generalization test of solar radiation under different weather conditions

**Figure 10:**
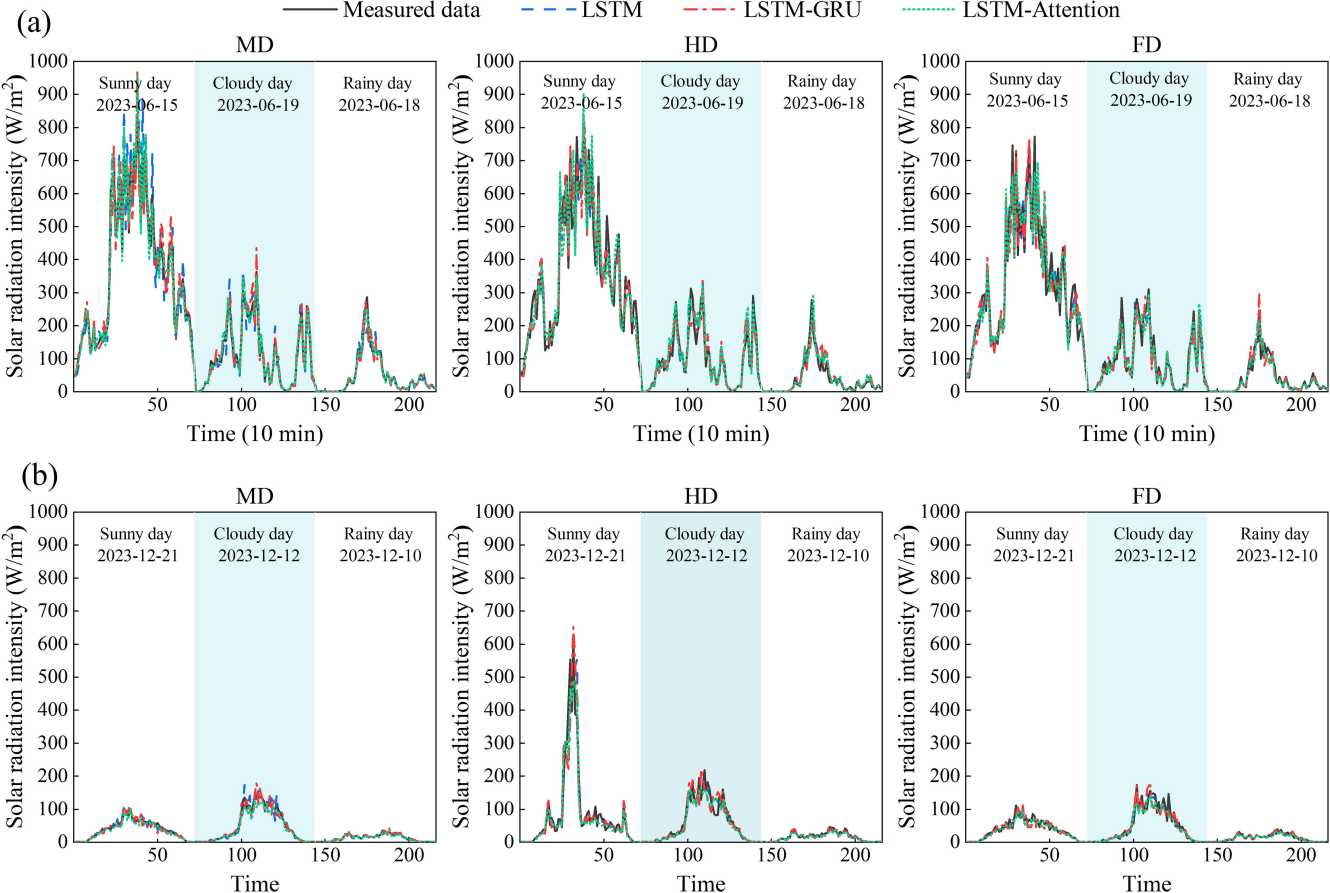
Prediction results of solar radiation intensity under different weather conditions in different seasons by LSTM, LSTM-GRU and LSTM-Attention models: **(a)** Summer; **(b)** Winter.

To partially evaluate the model’s generalization ability outside the training window, an additional validation was performed on a short period of data (3 days) not included in the training phase. This additional period, which was selected outside of both the summer and winter solstices, provided a preliminary check of the model’s robustness to conditions beyond the specific seasonal extremes. We evaluated its solar radiation forecasting performance relative to conventional LSTM and hybrid LSTM-GRU models under seasonally varied weather conditions (**Figure 10**). As shown in **Figure 10**, the mean maximum prediction deviations for the three models (LSTM-Attention, LSTM and hybrid LSTM-GRU models) under summer conditions were 139.2, 105.8, and 100.7 W/m^2^ for sunny days; 67.2, 58.6, and 41.2 W/m^2^ for cloudy days; and 44.7, 44.3, and 30.4 W/m^2^ for rainy days. Under winter conditions, the corresponding mean maximum deviations were 82.6, 68.2, and 5.6 W/m^2^ for sunny days; 47.1, 38.9, and 3.7 W/m^2^ for cloudy days; and 9.2, 8.3, and 1.0 W/m^2^ for rainy days. The comparative analysis of these curves and data demonstrated that, the LSTM-Attention model achieved superior prediction accuracy for solar radiation intensity compared to other models under different weather conditions.

**Table 2** presented a numerical comparison of how well the LSTM, LSTM-GRU, and LSTM-Attention models predicted solar radiation intensity in the AV system under typical seasonal weather. The LSTM-Attention model performed better than the others in all tested situations. For example, during summer clear-sky conditions at the MD treatment site, this model reached an RMSE of 31.8 W/m^2^ and an MAE of 25.7 W/m^2^. These values were 48.0% and 43.0% lower than those of the LSTM model. It also achieved an *R*^2^ of 97.6%. The advantage of the LSTM-Attention model was even clearer in cloudy and rainy weather. On rainy days, its MAE decreased to 41.6% of the LSTM model’s value. In winter clear sky, the LSTM-Attention model reduced the RMSE to 5.2 W/m^2^, improved by 39.5% compared with LSTM-GRU model. These results showed that the LSTM-Attention structure can adapt well to various weather situations.

**Table 2:** Comparison of model performance for solar radiation intensity under different weather conditions.

Season	Treatment	Weather type	LSTM			LSTM-GRU			LSTM-Attention		
			RSME/W·m^-2^	MAE/ W·m^-2^	*R*^2^/%	RSME/W·m^-2^	MAE/ W·m^-2^	*R*^2^/%	RSME/W·m^-2^	MAE/ W·m^-2^	*R*^2^/%
Summer	MD	Sunny day	61.1	45.1	91.3	44.1	32.1	95.5	31.8	25.7	97.6
		Cloudy day	25.7	17.2	92.2	20.7	14.4	94.9	14.4	10.0	97.5
		Rainy day	18.7	8.9	90.4	12.2	6.4	95.9	7.1	3.7	98.6
	HD	Sunny day	51.1	40.2	92.8	44.0	33.9	94.7	35.2	27.2	96.6
		Cloudy day	24.5	17.1	91.6	20.7	13.8	93.9	17.2	11.4	95.8
		Rainy day	16.1	8.9	91.0	12.8	6.9	94.4	8.3	4.4	97.6
	FD	Sunny day	49.1	39.2	92.2	39.3	31.1	95.0	29.3	22.4	97.2
		Cloudy day	23.0	16.5	91.3	20.1	12.5	93.4	14.0	9.9	96.8
		Rainy day	14.6	7.9	91.5	11.7	5.8	94.6	7.2	4.1	98.0
Winter	MD	Sunny day	39.6	22.8	92.3	8.6	5.8	94.6	5.2	3.5	98.0
		Cloudy day	20.2	11.1	91.4	12.5	7.8	93.6	10.2	6.0	95.7
		Rainy day	3.8	2.6	91.1	2.6	1.8	93.1	2.0	1.3	96.0
	HD	Sunny day	38.4	19.2	91.7	28.2	12.3	95.6	19.9	9.3	97.8
		Cloudy day	17.5	9.9	91.6	15.9	9.7	93.1	10.2	5.5	97.2
		Rainy day	3.4	2.4	91.9	2.9	1.9	93.9	2.5	1.6	95.7
	FD	Sunny day	11.0	6.1	91.2	8.2	5.8	95.1	5.6	3.6	97.7
		Cloudy day	14.4	8.6	91.6	12.5	7.5	93.7	9.5	5.6	96.3
		Rainy day	3.0	2.0	90.8	2.6	1.8	93.2	2.0	1.3	96.2

The LSTM-Attention model predicted the solar radiation intensity better than the LSTM-GRU model due to the attention mechanism that adjusted the importance of different weather features more efficiently. Although the LSTM-GRU model did not have much improvement in complex weather conditions, the LSTM-Attention model still maintained an *R*^2^ of 96.2% in winter rain, which was 5.4% higher than LSTM. It meant that it can deal with the non-linear effect of rainfall on radiation better. Additionally, as the difference among the three models became smaller with an increase in coverage density, LSTM-Attention still reduced the MAE by an average of 42.1% compared to LSTM under FD treatment. It showed that it works reliably in different circumstances. These findings indicated that hybrid models including attention mechanisms could greatly enhance the accuracy of solar radiation intensity prediction tasks, offering more dependable modeling tools for evaluating the performance of an AV system (Sakib et al., 2024).

### 4.3 Analysis of air temperature prediction results

#### 4.3.1 Seasonal variations in predicted air temperature

**Figure 11:**
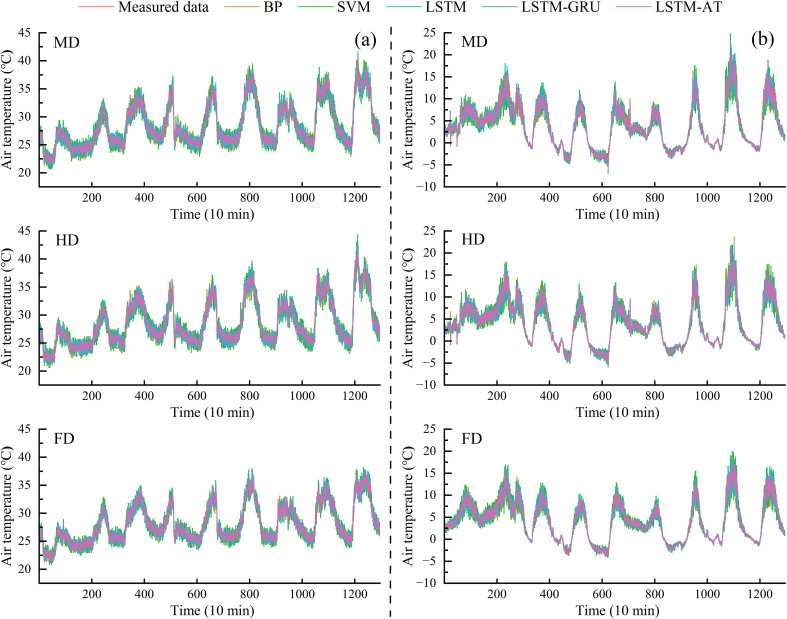
The estimated and measured value curve of air temperature: **(a)** Summer; **(b)** Winter.

**Figure 11** displayed comparative curves between predicted and measured air temperature values across different seasons for the BP, SVM, LSTM, LSTM-GRU, and LSTM-Attention models. The maximum prediction deviations for air temperature in summer under the MD, HD and FD treatment were 2.3, 1.6, 2.2, 1.8, and 1.0°C (MD); 2.3, 1.8, 2.3, 1.8, and 1.1°C (HD); and 2.9, 2.2, 1.6, 1.52, and 0.9°C (FD), respectively, for the five models. Correspondingly, the maximum prediction deviations in winter under the same coverage densities were 4.7, 4.3, 5.0, 3.7, and 3.8°C (MD); 6.7, 4.5, 4.7, 4.2, and 3.4°C (HD); and 7.5, 5.0, 4.3, 2.8, and 2.3°C (FD). The results showed that the LSTM-Attention model improved the ability of the model to forecast seasonal temperatures, with the prediction errors being much smaller than those of the traditional methods (BP, SVM) and other neural network structures (LSTM, LSTM-GRU). It is noteworthy that the curves predicted by the LSTM-Attention model were most consistent with the actual measurement data, especially showing better adaptability during periods of fast temperature changes. On the contrary, the BP and SVM models generated smoother prediction curves with less capacity to capture sudden temperature changes. Though the LSTM and LSTM-GRU models outperformed traditional ML techniques, they still had some lag or overshoot in their peak temperature predictions.

According to the error analysis, the LSTM-Attention model performed the best. In summer, under the MD treatment, the maximum deviation was only 1.0°C. It was much less than the deviations of the BP and SVM models, which were 2.3°C and 1.6°C, respectively. As the PV coverage density rose to the FD treatment, the maximum deviation of the LSTM-Attention model decreased further to 0.9°C. In contrast, the other models’ errors grew. In winter, the performance difference became even larger, with LSTM-Attention keeping a max deviation of 2.3°C for the FD treatment. The main reason for this performance improvement was that the attention mechanism could pay attention to the relevant parts of the temperature feature sequence, which improved the model’s ability to find the nonlinear relationship between the environment and temperature, so as to make more accurate predictions of temperature changes under complex environmental conditions. All models were impacted by the PV panel coverage density, but the degree of impact differed greatly. Traditional machine learning models (BP and SVM) had poor adaptability to environmental parameter changes as they showed significant performance deterioration when the coverage density increased. Deep learning models generally performed better, and LSTM-Attention was the most insensitive to changes in coverage density, indicating the best robustness. This observation indicates that for environmental temperature prediction tasks affected by the intensity of solar radiation, deep learning models with attention mechanisms can better deal with the differences in input features caused by different PV panel layouts, making them a more reliable prediction tool for real-world engineering applications, as reported by He et al (He et al., 2022).

Air temperature predictions showed different patterns under varying PV array densities. The temperature field within the AV system was heavily influenced by both solar radiation and shading effects. As the PV array density increased, the shading reduced the amount of direct sunlight reaching the ground, which in turn lowered the overall temperature. The model performed reasonably well in predicting air temperature in scenarios with the MD configuration. However, in the HD and FD configuration, the shading effects significantly altered the temperature distribution, leading to increased model errors, especially during periods of high solar radiation. The RMSE for air temperature predictions was higher in the HD configuration compared to the MD configuration, where temperature variations were more stable. This suggested that the model struggled to adapt to the complex temperature gradients caused by dense PV arrays. As a result, further adjustments were needed to enhance the model’s ability to capture such variations in high-density configurations.

**Figure 12:**
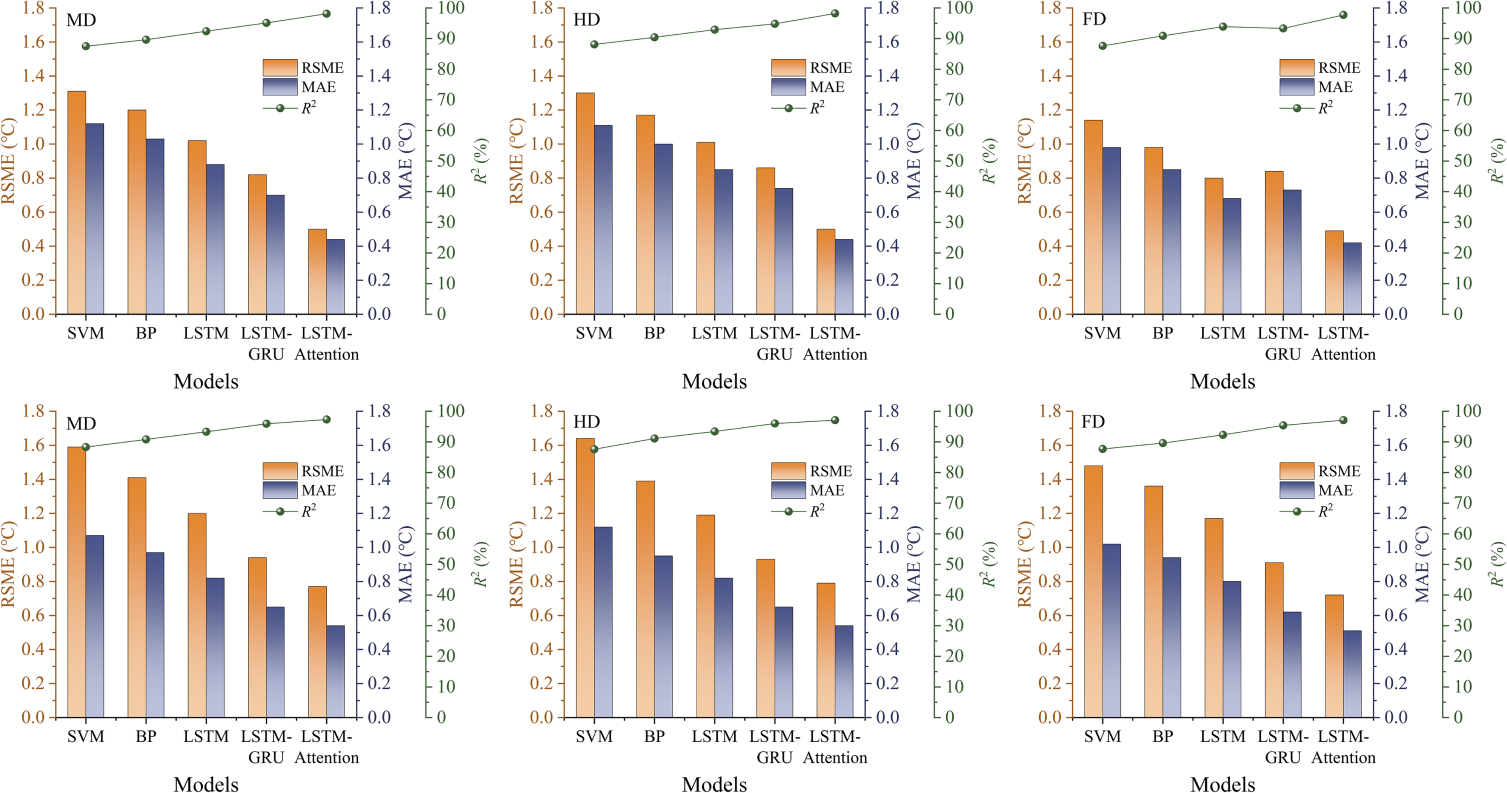
Performance comparison of five prediction models for air temperature in different forecasting seasons.

**Figure 12** showed the performance of five models in predicting air temperature in different seasons and PV panel coverage levels. LSTM-Attention model always did better than the rest no matter if it was summer or winter, and the coverage density didn’t matter. It has smaller RMSE and MAE values compared to LSTM-GRU and LSTM. For instance, in summer with the MD coverage, the LSTM-Attention model achieved an RMSE of 0.5°C. This was 39.0% lower than LSTM-GRU and 51.0% lower than LSTM. In winter at the same coverage level, it had an RMSE of 0.8°C, which was 18.1% and 35.8% less than LSTM-GRU and LSTM, respectively. The model also kept an *R*^2^ over 97.0% in all cases, indicating a strong capacity to explain temperature changes. Interestingly, in summer with HD coverage, the *R*^2^ was as high as 98.2%, which was much higher than those of LSTM-GRU and LSTM.

The improved accuracy of the LSTM-Attention model may have been because it had an attention mechanism that gave different weights to the input data. It helped the model to recognize the complex and changing temperature patterns usually seen in small climate areas inside AV systems. On the other hand, the LSTM-GRU model did not include a feature selection process, which may explain its larger variation in error. For instance, at the FD treatment site in winter, its RMSE was 0.9°C. Although this result was better than that of the standard LSTM model, it was still less accurate than LSTM-Attention. The basic LSTM model, affected by gradient vanishing problems, performed the worst when predicting long sequences. Its *R*^2^ value dropped to 92.3% in winter. These findings support the importance of using attention mechanisms to improve the accuracy of air temperature forecasts (Nandi et al., 2022).

#### 4.3.2 Out-of-window generalization test of air temperature under different weather conditions

**Figure 13:**
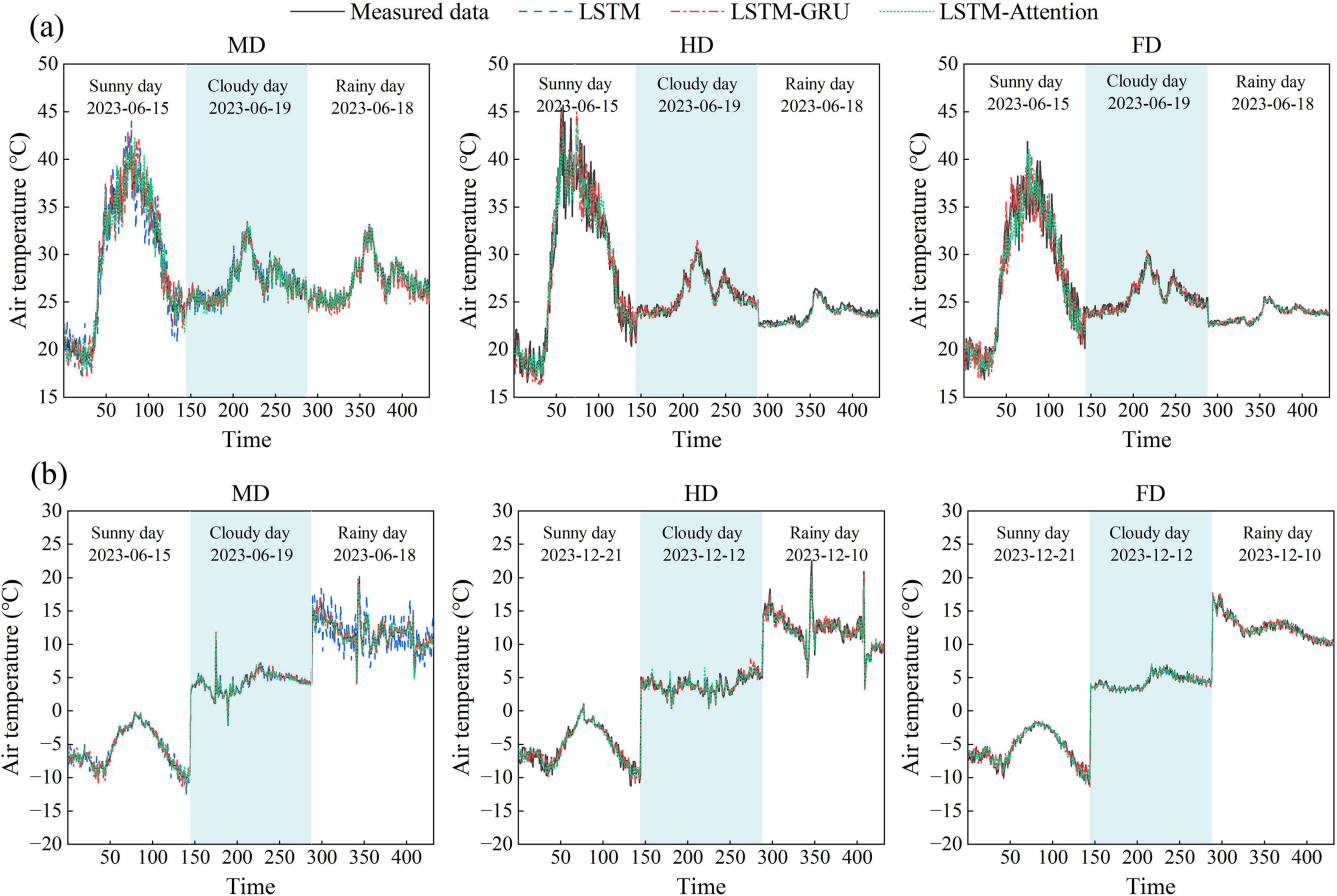
Prediction results of air temperature under different weather conditions in different seasons by LSTM, LSTM-GRU and LSTM-Attention models: **(a)** Summer; **(b)** Winter.

To partly assess how well the model performs outside the training period, we ran an extra validation using three days of data that were not part of the training set. These three days were chosen to avoid both summer and winter solstices. This helped us do an early check on whether the model could handle conditions that were not tied to the extreme seasons. We compared how three models (LSTM, LSTM-GRU, and LSTM-Attention) performed during summer and winter under three different types of weather: clear, cloudy, and rainy days (**Figure 13**). In summer, the highest average prediction errors on clear days were 3.9°C for LSTM, 3.5°C for LSTM-GRU, and 2.8°C for LSTM-Attention. On cloudy days, the errors dropped to 1.1°C, 0.8°C, and 0.6°C for the same models. For rainy days, the errors were even lower: 0.4°C for LSTM, 0.3°C for LSTM-GRU, and 0.2°C for LSTM-Attention. In winter, the maximum mean deviation on clear days was 1.8°C, 1.5°C, and 1.1°C; for cloudy days, it was 0.9°C, 1.0°C, and 0.9°C; and for rainy days, it was 2.2°C, 1.2°C, and 0.7°C for the above three models, respectively.

Performance comparison showed that the LSTM-Attention model had better performance than both GRU-combined and traditional LSTM models in terms of weather prediction accuracy. This improved performance was especially noticeable during summer clear-sky conditions, when the LSTM-Attention model cut down the prediction error by 27.7% compared to the regular LSTM model, and during winter rainy conditions, the improvement was as high as 67.7%. The stable performance under various weather conditions suggests that the attention mechanism can capture temperature changes affected by weather, which is difficult for traditional models to handle. In addition, during rainy conditions, the model showed higher prediction errors, likely due to rapid changes in radiation and temperature.

**Table 3** provided a quantitative comparison of air temperature forecasts in the AV system for different seasons by means of LSTM, LSTM-GRU and LSTM-Attention. The results indicated that the LSTM-Attention model performed better than the other models in terms of accuracy. In the summer clear sky situation at MD treatment, the RMSE of LSTM-Attention model was 1.4°C, which was 31.4% and 17.7% lower than the RMSE of LSTM and LSTM-GRU models, respectively. Its *R*^2^ was 95.9%, higher than the 92.1% and 93.9% of the other models. When it was overcast, this model did even better, with the RMSE dropping to 0.3°C and the *R*^2^ rising to 97.5%, indicating that it could do a better job capturing changes in air temperature under different lighting conditions. The LSTM-Attention model demonstrated especially strong stability during winter extreme weather events. Rainy condition at the MD treatment, it achieved an RMSE of 0.4°C, an 87.2% reduction from LSTM’s 3.1°C, and maintained a high *R*^2^ of 96.5%. Noteworthy is that in winter clear sky situation, the model’s MAE got as low as 0.4°C, which was 29.1% better than LSTM-GRU, proving the accuracy of detecting small changes in air temperature in cold places. This advantage became more obvious when there were more places covered.

**Table 3:** Comparison of model performance for air temperature under different weather conditions.

Season	Treatment	Weather type	LSTM			LSTM-GRU			LSTM-Attention		
			RSME/°C	MAE/°C	*R*^2^/%	RSME/°C	MAE/°C	*R*^2^/%	RSME/°C	MAE/°C	*R*^2^/%
Summer	MD	Sunny day	2.10	1.75	92.1	1.75	1.47	93.9	1.44	1.24	95.9
		Cloudy day	0.60	0.52	91.4	0.48	0.42	94.5	0.32	0.28	97.5
		Rainy day	0.31	0.28	91.3	0.29	0.26	92.3	0.27	0.23	93.4
	HD	Sunny day	2.10	1.70	93.0	1.79	1.54	94.9	1.29	1.05	97.3
		Cloudy day	0.49	0.42	92.0	0.48	0.42	92.4	0.30	0.26	97.0
		Rainy day	0.27	0.24	91.5	0.27	0.23	91.6	0.28	0.24	98.1
	FD	Sunny day	1.88	1.61	92.3	1.69	1.44	93.7	1.25	1.03	96.6
		Cloudy day	0.46	0.40	90.9	0.44	0.38	91.8	0.29	0.25	96.3
		Rainy day	0.22	0.19	91.4	0.21	0.19	91.9	0.14	0.13	96.4
Winter	MD	Sunny day	0.75	0.58	92.8	0.70	0.55	93.8	0.47	0.39	97.1
		Cloudy day	0.42	0.35	91.1	0.37	0.31	93.3	0.28	0.23	96.2
		Rainy day	3.05	1.98	92.7	0.61	0.53	91.1	0.39	0.32	96.5
	HD	Sunny day	0.80	0.61	91.4	0.61	0.47	94.9	0.47	0.38	97.0
		Cloudy day	0.57	0.47	91.5	0.54	0.46	92.4	0.34	0.28	97.0
		Rainy day	0.72	0.61	91.2	0.70	0.60	91.6	0.40	0.34	97.2
	FD	Sunny day	0.51	0.43	90.2	0.69	0.54	91.5	0.45	0.36	96.4
		Cloudy day	0.28	0.24	91.8	0.25	0.21	93.4	0.21	0.18	95.5
		Rainy day	0.65	0.52	92.5	0.48	0.40	91.3	0.28	0.24	96.9

In all kinds of weather conditions, LSTM-Attention always performed better than other models in complex meteorological situations. During the summer rainy season, *R*^2^ was high, ranging from 93.4% to 98.1%, which was higher than other models by 1.1% to 6.8%. In the winter overcast condition under HD treatment, the model got an MAE of 0.3°C, which was 40.4% lower than the baseline models, and an *R*^2^ of 97.0%. It can be seen that it can fit the non-linear relationship of air temperature under different weather conditions. The model consistently performed well throughout all seasons and weather conditions, showing that it can be relied upon to predict air temperature. From this, it can be seen that the LSTM memory retained longer context, which was essential for predicting delayed temperature responses. This mechanism based explanation was consistent with the observation that the gains of LSTM-Attention became more pronounced under complex weather, particularly in winter rainy scenarios.

### 4.4 Limitations and future work

This study presents some encouraging outcomes using the LSTM-Attention model for forecasting microclimate parameters in AV systems. However, there are some limitations that should be considered. A major limitation is that only one dataset from one particular area, under flat land conditions, was used. This might impact how well the model works in other places or under different terrain conditions. Therefore, future studies could consider validating the microclimate prediction model under various terrain conditions, such as sloping land. Additionally, although the attention mechanism enhances the model’s prediction accuracy, it has difficulty dealing with extreme weather conditions that have highly variable inputs. Future research will focus on optimizing the model to better handle such variations and improve its adaptability across different environments.

Despite these challenges, the results give us a good starting point to improve how we set up PV arrays and manage crops in AV systems. In the future, the proposed model can be integrated with streaming data from in-field sensors and weather stations to deliver rolling short-term forecasts, thereby supporting real-time AV management decisions such as irrigation scheduling and field operation planning. In addition, the model can be evaluated and applied across different PV coverage densities, and the forecasting module can be embedded into a management framework to enable quantitative co-optimization of PV array layouts and crop management under multi-objective trade-offs.

## 5 Conclusion

In this study, we developed a model based on LSTM and Attention mechanisms to predict microclimate such as solar radiation and air temperature in AV systems. The model was trained with information collected from the experimental AV systems on flat land in Nanjing, China. The results showed that the LSTM-Attention model performed much better than the traditional machine learning models (BP and SVM) and the standard LSTM model. Specifically, during the summer, the model decreased the RMSE of the solar radiation forecast by 28.0%, 35.7%, and 42.1% for the MD, HD, and FD treatments, respectively. In terms of air temperature prediction, the RMSE was reduced by 39.0% in summer and 18.1% in winter compared to the LSTM-GRU model. The attention mechanism allowed the model to focus on the most relevant temporal features, which improved its ability to detect complex changes in solar radiation and air temperature under different weather conditions such as sunny, cloudy, and rainy days. The model also performed well at both ends of the season. In winter, the RMSE for solar radiation predictions was 11.2 W/m^2^ and for air temperature predictions was 0.4°C under rainy conditions with the FD treatment. These results show that the LSTM-Attention model can do a good job at predicting microclimate parameters. This research provides a theoretical basis for the optimization of the array structure of AV systems and the adjustment of agricultural planting structure. At the same time, it offers important theoretical support for the microclimate prediction of AV projects in different regions. However, it should be noted that this study was only conducted under flat land conditions. Future studies can extend the model to different terrain types, such as sloping land, to further validate its applicability.

## Data Availability

The raw data supporting the conclusions of this article will be made available by the authors, without undue reservation.
